# An overview of clinical cerebral microdialysis in acute brain injury

**DOI:** 10.3389/fneur.2023.1085540

**Published:** 2023-02-21

**Authors:** Matthew G. Stovell, Adel Helmy, Eric P. Thelin, Ibrahim Jalloh, Peter J. Hutchinson, Keri L. H. Carpenter

**Affiliations:** ^1^Department of Neurosurgery, The Walton Centre, Liverpool, United Kingdom; ^2^Division of Neurosurgery, Department of Clinical Neurosciences, University of Cambridge, Cambridge, United Kingdom; ^3^Department of Clinical Neuroscience, Karolinska Institutet, Stockholm, Sweden; ^4^Department of Neurology, Karolinska University Hospital, Stockholm, Sweden; ^5^Department of Clinical Neurosciences, Wolfson Brain Imaging Centre, University of Cambridge, Cambridge, United Kingdom

**Keywords:** cerebral microdialysis, traumatic brain injury (TBI), subarachnoid hemorrhage (SAH), neurocritical care, brain injury, cerebral physiology

## Abstract

Cerebral microdialysis may be used in patients with severe brain injury to monitor their cerebral physiology. In this article we provide a concise synopsis with illustrations and original images of catheter types, their structure, and how they function. Where and how catheters are inserted, their identification on imaging modalities (CT and MRI), together with the roles of glucose, lactate/pyruvate ratio, glutamate, glycerol and urea are summarized in acute brain injury. The research applications of microdialysis including pharmacokinetic studies, retromicrodialysis, and its use as a biomarker for efficacy of potential therapies are outlined. Finally, we explore limitations and pitfalls of the technique, as well as potential improvements and future work that is needed to progress and expand the use of this technology.

## 1. Introduction

**Figure 1:**
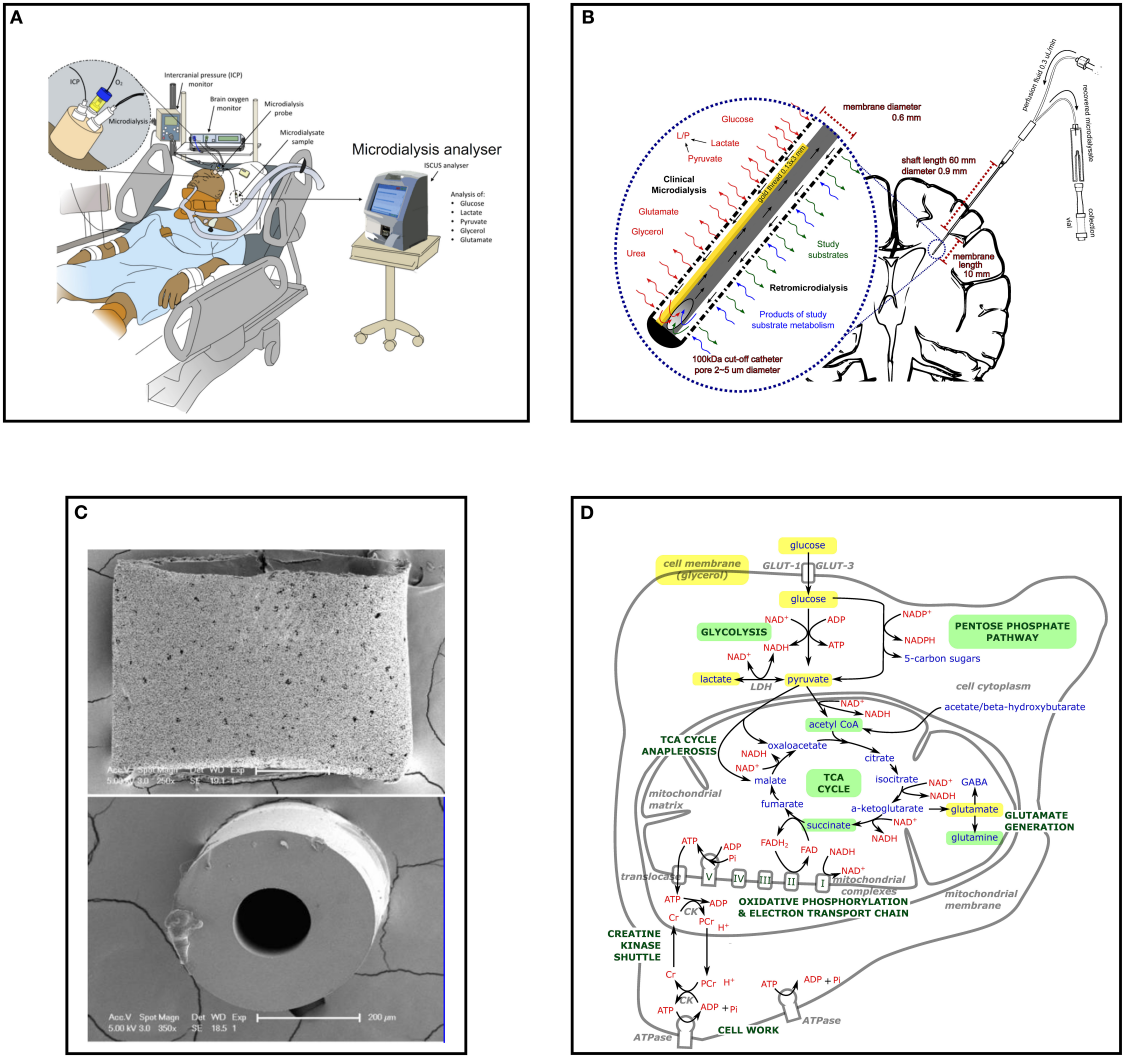
Cerebral microdialysis from macroscopic to microscopic. **(A)** Multimodality monitoring in neurocritical care; Illustration of neurocritical care multimodality monitoring including intracranial pressure (ICP), brain tissue oxygen (PbtO_2_) and microdialysis with an ISCUS/ISCUS*flex* which are employed alongside standard intensive care monitoring of systemic physiology. Illustration copyright Susan Giorgi-Coll and reproduced here with her permission. **(B)** Schematic of cerebral microdialysis; 100 kDa cut-off microdialysis catheter inserted into patient's brain, with magnified view of catheter tip demonstrating semipermeable membrane. A gold filament (0.13 x 3 mm) along the end of the catheter allows its identification on CT scans and some MRI sequences. The microdialysis perfusate is pumped slowly (0.3 μL/min) along the outer lumen, the final 10 mm of which is a semipermeable membrane (100 kDa cut-off, outer pore diameter ≈ 2–5 μm). Small molecules freely exchange with the brain interstitium, approaching equilibrium by the time they reach the recovery hole in the end of the central, non-porous channel, where the fluid passes back for collection in microdialysis vials. Glucose, lactate, pyruvate, glycerol, glutamate and urea may be used for clinical monitoring, illustrated to the left of the expanded window. Catheters can also be used to “dose” the surrounding brain with research substrates (including those with stable isotope ^13^C labeling) and recover the resultant products, illustrated to the right of the expanded window. **(C)** Scanning electron micrograph of M Dialysis 71 microdialysis catheter sectioned transversely; Upper panel the catheter semipermeable membrane (polyarylethersulfone) external surface demonstrating pores several micrometers across. **(B)** The central polyurethane non-porous inner channel removed from inside a catheter. Originally published in Helmy et al. (2). Copyright Mary Ann Liebert, Inc. and reproduced with permission (2). **(D)** Schematic of stylized cell with (enlarged) central mitochondrion illustrating principal metabolites and pathways of energy metabolism examined by cerebral microdialysis. Glucose, pyruvate, and lactate are important species in glycolysis and the TCA cycle for energy metabolism and the regeneration of ATP in conjunction with oxidative phosphorylation and the electron transport chain. Glutamate is the principle excitatory neurotransmitter in the brain and glycerol is a key component of cell membranes. All can be interrogated with clinical microdialysis (highlighted yellow). Glycolysis, the pentose phosphate pathway, the TCA cycle and succinate and glutamine metabolism have been investigated in research studies using microdialysis and retromicrodialysis (highlighted light green). GLUT, glucose transporter; NAD^+^/NADH, nicotinamide adenine dinucleotide; ADP, adenosine diphosphate; ATP, adenosine triphosphate; Pi, inorganic phosphorous; PCr, phosphocreatine; Cr, creatine; NADP+/NADPH, nicotinamide adenine dinucleotide phosphate; LDH, lactate dehydrogenase; TCA, tricarboxylic acid.

Cerebral microdialysis is an invasive monitoring technique used in neurocritical care (Figure 1A) that allows continuous monitoring of cerebral metabolism in patients who have sustained a significant brain injury such as a severe traumatic brain injury or poor-grade subarachnoid hemorrhage (1). Microdialysis also has important research applications that include the characterization of neuroinflammation, assessment of potential neuroprotective drugs, and direct intraparenchymal substrate delivery through retromicrodialysis/retrodialysis. An overview of cerebral microdialysis catheter structure and insertion, interpretation of results in clinical care, identification on imaging, research applications, limitations of the technique and likely future developments are described.

## 2. Catheter structure and setup

Cerebral microdialysis catheters consist of a dual lumen channel-within-a-channel (Figures 1B, C). The terminal 10 mm outer wall is a semi-permeable membrane through which small molecules can diffuse. As fluid (perfusate) is slowly pumped down the catheter and along the semi-permeable membrane, it exchanges solutes and fluid with the surrounding brain interstitium. When fluid reaches the catheter's closed tip it is forced to return *via* an inner non-porous central channel where it is ultimately recovered for analysis. Dedicated microdialysis pumps drive the perfusion fluid slowly enough that the concentration of metabolites in the recovered fluid (microdialysate) has time to approach equilibrium with that of the brain extracellular space. A variety of catheters exist that are designed for different tasks and tissue types; possessing varying shaft length, membrane length and membrane pore size. Currently cerebral microdialysis catheters with a membrane cut-off of < 20 kDa (M Dialysis 70 catheter) and < 100 kDa (M Dialysis 71 High Cut-Off catheter) are commercially available (M Dialysis AB, Stockholm, Sweden).

Microdialysis pumps may have fixed (M Dialysis 106) or variable (user-adjustable) flow rates (M Dialysis 107). A flow rate of 0.3 μL/min is considered standard for cerebral studies, as higher perfusion rates cause greater net fluid loss and incomplete equalization of microdialysate metabolite concentration from the surrounding brain (1, 3). “Standard” perfusion fluid used for cerebral microdialysis is crystalloid consisting of an aqueous solution of NaCl (147 mmol/L), KCl (2.7 mmol/L), CaCl_2_ (1.2 mmol/L), and MgCl_2_ (0.85 mmol/L), and is available commercially as a sterile ready-made solution (Perfusion Fluid CNS, from M Dialysis AB) but alternatives including those with the addition of dextran, or a human albumin solution can be used as fluid carriers (2, 4). Recently, a ready-made sterile solution consisting of ‘standard' perfusion fluid supplemented with 3% Dextran 500 kDa (Perfusion Fluid CNS Dextran, from M Dialysis AB) has become commercially available which has been shown to improve protein recovery *in vitro* (5).

## 3. Catheter insertion

Cerebral microdialysis catheters can be inserted during surgery if a patient requires a craniotomy or craniectomy, or *via* a single burr hole (often supported by a cranial access device)–either independently or alongside other multimodality monitoring such as brain tissue oxygen tension (PbtO_2_), intracranial pressure (ICP) or intraparenchymal temperature monitoring (1). Catheters must be inserted with their whole membrane within the brain parenchyma (typically white matter centrum semiovale). In diffuse TBI and SAH the non-dominant (right) frontal lobe is favored, at or anterolateral to Kocher's point (6). In more focal TBI or SAH with asymmetric hemorrhage or a region identified to be at risk of delayed cerebral ischemia, catheters may be directed ipsilateral to such lesions whilst avoiding eloquent areas and non-viable regions of injury. Targeting stroke penumbra or TBI peri-lesional border zones as areas of greatest risk that potentially will benefit most from monitoring and optimizing therapy is not easy to achieve, and if located too close to a lesion the detected metabolic pattern can be difficult to interpret clinically (1). Furthermore, extrapolating physiology from peri-contusional regions to “radiologically normal” brain is challenging, so multiple catheters may be employed, but this in turn makes interpretation of results complex.

## 4. Sampling and bedside analysis

Cerebral microdialysis catheters perfusing at 0.3 μL/min will produce ca. 18 μL of recovered microdialysate in an hour. Routinely, this is collected in dedicated micro-vials that are changed manually and analyzed hourly by the bedside using an enzymatic colorimetric analyzer, such as a CMA 600, ISCUS, or ISCUS*flex* (M Dialysis AB). Glucose, lactate, pyruvate, glycerol, glutamate and urea can be measured in this way, with results from analysis representing cerebral physiology from the previous hour of perfusion, as well as the ca. Twenty-minute time-lag for the fluid to return *via* the distal channel. As each metabolite only consumes 0.2–1.5 μL of microdialysate, a significant volume usually remains for further research applications of individual vials, or their pooled microdialysates.

Recent introduction of continuous on-line analyzers such as the MD System Loke (M Dialysis) use coated electrode biosensors that analyze the recovered microdialysate at greater frequency without the need for collection vials. A drawback of this system is its current inability to measure pyruvate and therefore inability to calculate lactate/pyruvate ratio (LPR), and its inability to collect the surplus microdialysate fluid for additional research use.

After insertion of a catheter, a very high initial rate of perfusion (15 μL/min) is automatically run by the microdialysis pump as a “flush” sequence for the first 5 min to expel the air from inside the catheter. This recovered fluid is discarded, as it will have had insufficient time to approach equilibrium with the surrounding brain. The subsequent results from the first hour of monitoring after catheter insertion should similarly be discarded to avoid transient insertion artifacts from cerebral micro-injury caused by catheter insertion.

## 5. Metabolites analyzed in clinical care

Cerebral microdialysis allows patients' brain extracellular chemistry to be continually monitored, and attempts made to tailor treatment to patients' cerebral metabolism. Glucose is considered the human brain's principal metabolic fuel, and extremes of high and low microdialysate glucose are associated with worse outcome after TBI and SAH (1, 7, 8). By monitoring brain glucose, critical neuroglycopenia can be detected and attempts made to reverse it, such as with infusion of intravenous glucose (1) or loosening of glycemic control (9, 10). Low cerebral glucose after SAH may also herald impending delayed cerebral ischemia, together with an elevated LPR and glutamate, allowing pre-emptive intervention (8).

A patient's cerebral LPR is perhaps the most important microdialysis biomarker as it reflects the NADH/NAD^+^ redox status (11) of the brain–the balance between aerobic and anaerobic metabolism–and a value ≥ 25 (or ≥ 40 in some centers) in radiologically “healthy” brain is an independent predictor of poor patient outcome (7, 12). Furthermore, in the presence of low brain pyruvate and low PbtO_2_, a high LPR suggests brain ischemia, which may be treated by increasing cerebral perfusion pressure and inspired oxygen concentration. However, if LPR is high in the presence of a normal brain glucose concentration, normal cerebral perfusion pressure, and normal or high brain pyruvate and PbtO_2_, the patient's brain is likely suffering from cellular mitochondrial dysfunction, for which there is currently no universally recognized treatment (1, 13–16). To allow differentiation of hypoxic elevation of LPR from primary mitochondrial dysfunction, cerebral microdialysis is preferably used together with brain tissue oxygen tension (PbtO_2_) monitoring and intracranial pressure monitoring from an equivalent region of brain Figure 1A.

High brain glutamate concentration is thought to represent harmful excitotoxicity (1, 17, 18), but the concentration in the extracellular space may often be under the detection limit of bedside ISCUS analysis, so its results may be less reliable. Nonetheless, it is associated with poor outcome in large studies of TBI and SAH (7, 8).

The poor specificity of glycerol makes its significance even more questionable as although it may represent cell membrane breakdown from oxidative stress (19), it is also a common metabolic intermediate of glucose (20, 21) and is even found in some of the drugs used in the treatment of patients in neurointensive care, although these do not seem to affect glycerol levels (22).

Microdialysis measurements of glucose, lactate, pyruvate and LPR are thus often considered more important markers of metabolism than measurements of glutamate and glycerol (1), and urea is principally used as an endogenous reference compound (23). The key relationship between cellular energy metabolites measured using cerebral microdialysis are illustrated in Figure 1D.

## 6. Identification on imaging sequences

**Figure 2:**
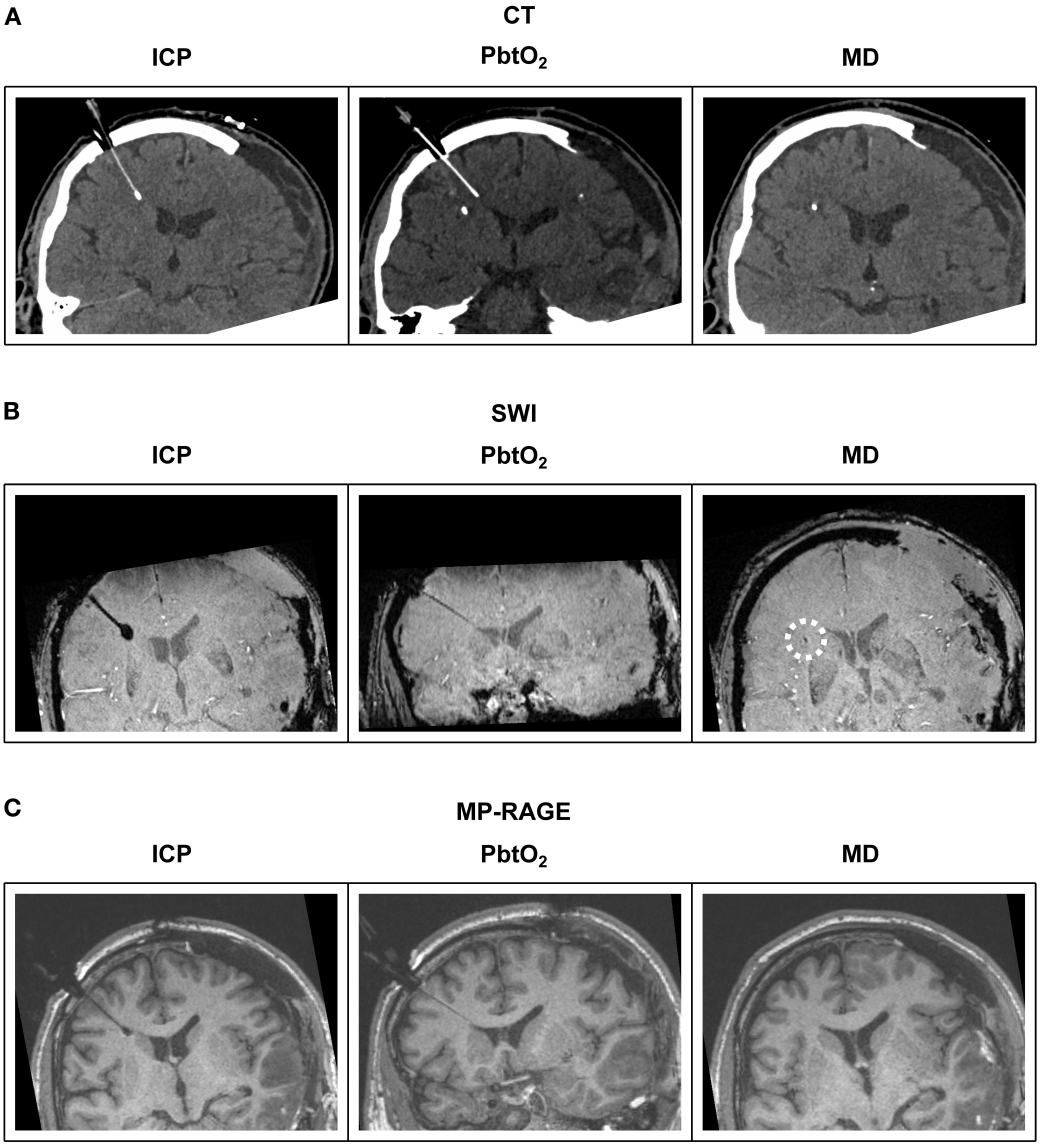
Illustrative examples of multimodal monitoring appearance on CT and MRI. Reformatted coronal slices demonstrate ICP, PbtO_2_ and microdialysis probes in a patient's brain. **(A)** CT; The ICP probe's thin shaft and bulbous end can be distinguished from the PbtO_2_ probe's uniform shaft and tip. Only the gold filament at the end of the microdialysis catheter is visible. **(B)** MRI T2-weighted SWI sequence; The same bulbous tip of the ICP probe and uniform thickness PbtO_2_ probe is revealed as with CT. The gold tipped microdialysis catheter is just visible on SWI sequences (highlighted by white dashed circle). **(C)** MRI T1-weighted MP-RAGE sequence; The same bulbous tip of the ICP probe and uniform thickness PbtO_2_ probe is revealed as with CT and SWI. The gold tipped microdialysis catheter is not visible. CT, computed tomography; SWI, susceptibility weighted imaging; MP-RAGE, T1-weighted magnetization-prepared rapid gradient-echo; ICP, intracranial pressure; PbtO_2_, brain tissue oxygen tension; MD, microdialysis.

Cerebral microdialysis catheters contain a short gold filament within their tip which is visible on computerized tomography (CT) and some magnetic resonance imaging (MRI) sequences (Figure 2). Microdialysis catheters are otherwise poorly visible on these imaging modalities, unlike intracranial pressure and brain tissue oxygen probes. As microdialysis pump batteries are not MRI compatible/conditional, the pump must be disconnected from the catheters during an MRI scan, but the catheters themselves can remain *in-situ* for reconnection after the patient has been removed from the scanner.

## 7. Research applications including retro-microdialysis

Observational studies analyzing the results of cerebral microdialysis from large patient cohorts have identified an association between patient outcome and bedside microdialysis measurements of cerebral energy species glucose and LPR (and glutamate), supporting clinical management of these patients (7, 12).

Neuroinflammation is an important contributor to secondary brain injury after TBI and SAH; involving microglial activation, attraction of circulating macrophages, astrocyte activation and production of cytokines (24–28). Microdialysis has revealed complex interactions of cytokines, chemokines, interferons, interleukins and tumor necrosis factors in human TBI (29), with increases in MCP1, MCP 2, CXCL10, *IL-1b*, IL6, and IL8 corroborated across different studies (24, 28, 29), potentially associated with outcome and metabolic stress. Small protein cytokines are best recovered with M Dialysis 71 High Cut-Off catheters (nominal molecular weight cut-off 100 kDa) and human albumin solution or dextran as a carrier (2, 24, 28), then measured with dedicated assays that may require amplification due to their low concentrations (28).

Cerebral microdialysis can also be used to measure the concentration of systemically administered drugs and study substrates to characterize their cerebral pharmacokinetics, including blood-brain-barrier penetration (30–33). The effect of neurotherapeutic agents or interventional procedures can also be assessed through changes in cerebral energetics measured using bedside clinical analysis, or with more advanced off-line methods (3, 34, 35). Using cerebral microdialysis to assess pharmacokinetics and pharmacodynamics is currently under-utilized in Phase II clinical trials (36).

Study substrates can be added to microdialysis perfusion fluid, and *via* retromicrodialysis/retrodialysis, small regions of brain can be micro-dosed focally with agents that further interrogate the metabolism of the brain (37, 38), and/or attempt to ameliorate cellular metabolic crises (39–41). Whereas, it is assumed that study substrates with molecular weights smaller than the nominal molecular weight cut-off of the catheter's membrane will diffuse into the surrounding brain, it is not clear how far into the brain this diffusion effectively reaches, nor at what concentration. Nonetheless, assuming similar diffusion properties, the recovered fluid originates from the same or similar volume of brain perfused *via* retrodialysis so should represent the region effectively “dosed” by the study substrate.

Labeling study substrates with ^13^C allows the fate of the study molecule to be ascertained from identifying the position that the ^13^C ends up in within recovered metabolites with high-resolution nuclear magnetic resonance spectroscopy or mass spectrometry (37, 42–44). Due to limitations of sample volumes and sensitivity, fluid from multiple hours of microdialysate vials may need to be pooled together, depending on type of analysis used.

## 8. Limitations and potential issues

The 0.6–0.9 mm diameter channel-within-a-channel concentric design of microdialysis catheters makes them inherently fragile; even more so than ICP and PbtO_2_ probes. When inserted through craniostomies or cranial access devices (triple-bolts), catheters may fail to pierce the brain's dura, arachnoid or pia mater so the extradural, subdural or subarachnoid space is monitored instead of the brain parenchyma. This may not initially be apparent, so microdialysis catheter location should be noted on subsequent patient CT scans. Similarly, if results from microdialysis do not concur with other multimodality monitoring, a CT scan should be arranged to confirm catheter position, particularly if these results may influence treatment. Furthermore, unlike static ICP and PbtO_2_ monitors, cerebral microdialysis requires continuous pumping of fluid past a delicate semipermeable membrane sited in living tissue. Pump batteries can fail or be depleted, and pumps may fail mechanically. Within the brain a degree of infiltration of cells into the semipermeable membrane occurs and potentially partially obstructs the pores (2). Vials must be changed hourly and analyzed when using a CMA600/ISCUS/ISCUS*flex* analyzer over many days of monitoring. If the operator is busy with other aspects of patient care, changing of vials may be neglected or any of the problems with perfusion described above not identified. CMA600 or ISCUS*flex* analyzers can analyze multiple patients using a single machine, which unfortunately can result in vials being inserted in the wrong vial positions in the machine, which will cause erroneous results or may cause machine failure/software crash due to detection of unexpected vials. Newer continuous on-line analyzers (MD System Loke) may avoid some of these issues but are disadvantaged by their lack of pyruvate detection at present and thus calculation of LPR is unavailable. Microdialysis catheters may also be accidentally dislodged during patient transfer or during general nursing care. Erroneous results from microdialysis failure occurring intermittently can be excluded from clinical or research analysis without significant impact, but long periods of failed monitoring may significantly hinder treatment or data analysis.

Microdialysis is a focal monitoring technique measuring cerebral physiology of its immediate vicinity. Clinically this is often extrapolated to be an estimation of a patient's whole brain metabolism, but this may not be accurate given known regional variations in cerebral metabolism after TBI and SAH (45, 46). Furthermore, it remains unclear what can or should be done to treat the spectrum of perturbations to cerebral physiology that may occur, when they are observed.

## 9. Looking to the future

The adoption of cerebral microdialysis is currently limited to mainly academic neurointensive care centers, with only 30 across Europe and the USA using it for routine patient care (M Dialysis). For it to progress into a tool that is considered “standard” for the management of patients with severe acute brain injury in intensive care; firstly, specific metabolic abnormalities must be better defined in individual patient cohorts (12). Interventions that address these abnormalities in specific patient groups must then be developed into chemistry-driven protocols and agreed on (36, 40), before testing in multicenter clinical trials like other multi-monitoring modalities (47, 48). Expansion of microdialysis would also be supported by improvements in both the robustness and user-friendliness of its hardware, automation of bedside analysis and on-line continuous monitoring (49, 50), as well as lower cost of consumables.

## 10. Conclusions

Microdialysis has become an established component of multimodality monitoring that can provide continuous assessment of cerebral metabolism after severe acquired brain injury from trauma or stroke. Its powerful research applications include assessment of pharmacokinetics and pharmacodynamics of novel neurotherapeutics, characterization of neuroinflammation, and retrodialysis delivery of drugs to the injured brain. Recent advances in analytical technology have enlarged the scope of microdialysis, making it a powerful tool for clinical research. In terms of routine use of microdialysis in neurocritical care, there is still much scope for making microdialysis more user-friendly and less demanding on staff time. Furthermore, the utility of microdialysis in management and therapy for critical neurological injury could be increased by establishing treatment protocols for recognized derangements of cerebral physiology.

## Author contributions

MS: concept of the manuscript and writing original draft of the manuscript. MS and AH: creation of images. MS, AH, ET, IJ, PH, and KC: review and editing of the manuscript. All authors contributed to the article and approved the submitted version.
